# Is there a shared neurobiology between aggression and Internet addiction disorder?

**DOI:** 10.1556/JBA.3.2014.1.2

**Published:** 2014-03-24

**Authors:** Changtae Hahn, Dai-Jin Kim

**Affiliations:** Department of Psychiatry, Seoul Saint Mary’s Hospital, College of Medicine, Catholic University of Korea, Seoul, Republic of Korea

**Keywords:** Internet, addiction, neurobiology, aggression

## Abstract

*Purpose:* Evidences indicate that Internet addiction disorder (IAD) has a higher risk of developing aggression and violent behavior. A few correlation studies between IAD and aggression have implicated a common biological mechanism. However, neurobiological approaches to IAD and aggression have not yet been studied. *Methods:* A literature search for studies for Internet addiction disorder or aggression was performed in the PubMed database and we selected articles about neurobiology of IAD or aggression. *Results:* This review includes (a) common neural substrates such as the prefrontal cortex and the limbic system between aggression and IAD; (b) common neuromodulators such as dopamine, norepinephrine, serotonin, opiate and nicotine between aggression and IAD. *Conclusions:* Through reviewing the relevant literature, we suggested the possibility of common neurobiology between the two psychiatric phenomena and direction of research on aggression in IAD.

## Introduction

### Internet addiction disorder

Use of the Internet in modern society has rapidly increased in recent years. Since the Internet is extensively used in academic, recreational and business sectors, the relationship between the Internet and modern people has become truly inseparable. Although the Internet functions in a positive way for people to enjoy leisure, explore information, and expand interpersonal relationships, a loss of control over excessive Internet use has been proposed as a pathological problem named Internet addiction disorder (IAD) (Ko, Yen, Chen, Chen & Yen, 2005; Ko, Yen, Yen, Chen & Chen, 2012; Shapira et al., 2003; Young, 1998). Loss of control over excessive Internet use is considered as an addictive disorder by a number of researchers (Alavi, Maracy, Jannatifard & Eslami, 2011; Dong, Lu, Zhou & Zhao, 2011; Dong, Zhou & Zhao, 2010; Lin et al., 2012; Liu et al., 2010; Orzack & Orzack, 1999; Tonioni & Corvino, 2011; Xiuqin et al., 2010; Yuan et al., 2011; Zhu, Jin & Zhong, 2009; Zhu, Jin, Zhong, Chen & Li, 2008; Zhu, Li, Du, Zheng & Jin, 2011). Although official diagnostic criteria have not been established for IAD, four essential factors have been suggested (Block, 2008): 1) excessive Internet use, 2) withdrawal symptoms, 3) tolerance and 4) adverse consequences. Thus, IAD was considered to be included in the category of substance use and addictive disorders in the Diagnostic and Statistical Manual of Mental Disorders-5 (DSM-5).

### Psychiatric problems of Internet addiction disorder

Several researchers have suggested comorbidity between IAD and psychiatric symptoms (Ko, Yen, Chen, Yeh & Yen, 2009; Ko et al., 2012; Shaw & Black, 2008; Weinstein & Lejoyeux, 2010). A number of papers showed that IAD is closely associated with alexithymia (De Berardis et al., 2009), hostility (Ko, Yen, Yen, Lin & Yang, 2007; Shapira et al., 2003; te Wildt, Putzig, Zedler & Ohlmeier, 2007; Yen et al., 2008), aggressive behavior (Ko, Yen, Liu, Huang & Yen, 2009), impulsivity (De Berardis et al., 2009), dissociative symptoms (Bernardi & Pallanti, 2009; De Berardis et al., 2009), substance use disorders (Bai, Lin & Chen, 2001; Ko, Yen, Yen et al., 2008; Korkeila, Kaarlas, Jaaskelainen, Vahlberg & Taiminen, 2010; Lam, Peng, Mai & Jing, 2009; Yen, Ko, Yen & Chen, 2009), low self-esteem (De Berardis et al., 2009), affective disorders [dysthymic disorder (Bernardi & Pallanti, 2009; Ko, Yen & Chen, 2008), depression (Kim et al., 2006; Ko, Yen & Chen, 2008; Kraut et al., 1998; Lee et al., 2008; te Wildt et al., 2007; Yen et al., 2008; Yen, Ko, Yen, Wu & Yang, 2007), hypomania (Bernardi & Pallanti, 2009), bipolar I disorder (Shapira, Goldsmith, Keck, Khosla & McElroy, 2000)], anxiety disorders [social anxiety disorder (Bernardi & Pallanti, 2009; Ko, Yen & Chen, 2008; Ko, Yen & Chen et al., 2009; Yen et al., 2007), general anxiety disorder (Bernardi & Pallanti, 2009)], personality disorders (obsessive compulsive personality disorder, borderline personality disorder, avoidant personality disorder) (Bernardi & Pallanti, 2009), suicidal ideation (Kim et al., 2006), obsessive compulsive symptoms (Ha et al., 2006) and attention-deficit hyperactivity disorder (Bernardi & Pallanti, 2009; Ha et al., 2006; Ko, Yen & Chen, 2008; Ko, Yen, Chen et al., 2009; Yen, Yen, Chen, Tang & Ko, 2009; Yoo et al., 2004). Among the above psychiatric symptoms, the present article would like to discuss aggression and violence related with IAD.

### Aggression and violence in Internet addiction disorder

Aggression is a prevalent phenomenon with considerable cost in our society. The harmful effects of aggression are reported in the media on a daily basis and people can recognize the social significance of aggression through the media. Ironically, an estimated 10% to 30% of violence in society can be attributed to the impact of media violence, although it is debatable whether the media is a significant factor of violence (Strasburger, 2007). Among various forms of modern media, one of the most influential forms is the Internet.

Aggression can be classified into two types. The first type is premeditated aggression, which represents a planned behavior, also named as predatory, instrumental, or proactive aggression (Barratt & Felthous, 2003; Blair, 2004; Meloy, 2006; Siever, 2008). The second type is impulsive aggression, which is highly related with autonomic arousal and precipitation by provocation associated with negative emotions such as anger or fear (Blair, 2004; Meloy, 2006; Siever, 2008). Impulsive aggression is also referred to as reactive, affective or hostile aggression (Siever, 2008). Neurobiological and psychopathological mechanisms of impulsive aggression have been actively researched and understood. Therefore, we would like to focus our discussion on impulsive aggression in IAD.

Several studies have reported that aggression and violence are associated with IAD (Table 1) (Alavi et al., 2011; Dong, Lu et al., 2011; Ko, Yen, Liu et al., 2009; Mehroof & Griffiths, 2010; Yen et al., 2007; Yen, Yen, Wu, Huang & Ko, 2011; Zboralski et al., 2009). However, aggression and violence in IAD cannot be solely explained by addictive symptoms such as withdrawal or tolerance. In addition, neurobiological mechanisms have not yet been elucidated. This is not surprising because, presently, little is understood regarding the neurobiology of IAD. Many studies have started to suggest that IAD is an abnormality in the structure and function of the brain. Several investigators have researched structural changes in IAD by neuroimaging techniques. They have reported that patients with IAD have white matter integrity changes in brain regions such as thalamus, cingulate, cingulum, orbitofrontal, corpus callosum, fronto-occipital fasciculus, corona radiation, internal and external capsule and parahippocampal gyrus (Dong, Devito, Huang & Du, 2012; Lin et al., 2012; Yuan et al., 2011). Several studies also reported volume reduction of gray matter in dorsolateral prefrontal cortex, orbitofrontal cortex, cerebellum, supplementary motor area, cingulate cortex, insula and lingual gyrus are observed in patients with IAD (Yuan et al., 2011; Zhou et al., 2011). However, the results were rather inconsistent. Therefore, more studies are needed to clarify whether these changed structures are key neural substrates of IAD. On the neuropharmacological level, neuromodulators such as dopamine, norepinephrine, serotonin and acetylcholine may be related with the development of IAD (Han, Hwang & Renshaw, 2010; Han et al., 2007; Hou et al., 2012; Kim et al., 2011; Lee et al., 2008; Montag, Kirsch, Sauer, Markett & Reuter, 2012; Zhu et al., 2008).

Several studies indicated that neurobiology of aggression and violence could be explained by dysfunctions of brain circuitry, neurotransmitters, neuropeptides or genes (Siever, 2008). These studies suggested that there might be a common neurobiological mechanism between IAD and aggression. Thus, this article would like to elaborate on the close relationship between aggression and IAD and reviewing relevant studies.

## Methods

A literature search for studies for IAD or aggression was performed in the PubMed database using the search terms “Internet AND (addict* OR ((excessive OR pathological) AND use))” and “aggression OR hostili*”. Then we selected articles on neurobiology of IAD or aggression. Articles that had been published in English before October 2013 were included. Additional publications which were not found in the original search were complemented by reviewing reference lists of all retrieved studies.

**Table 1.** Review of aggression and Internet addiction disorder

Author	Year	Sample (*n*)	Aggression assessment	
Alavi et al.	2011	250	Symptom Checklist-90-Revision	There was an association between aggression and Internet addiction.
Ko, Yen, Liu et al.	2009	9405	Adolescent Aggressive Behaviors Questionnaire	Adolescents with Internet addiction were more likely to have aggressive behaviors during the previous year.
Yen et al.	2007	2114	Chinese Hostility Inventory – Short Form	Hostility is associated with Internet addiction in male adolescents.
Yen et al.	2011	2348	Durkee Hostility Inventory – Chinese version – Short Form	Subjects with Internet addiction had higher hostility and increased expressive hostility behavior.
Zboralski et al.	2009	120	Psychological Inventory of Aggression Syndrome	More frequent use of the computer and the Internet was connected with higher levels of aggression.
Dong, Lu et al.	2011	59	Symptom Checklist-90	Hostility could be the outcome of Internet addiction disorder.
Mehroof & Griffiths	2010	123	Buss Perry Aggression Questionnaire	The positive association between aggression and online gaming addiction.
Holtz et al.	2011	205	Youth Self Report	Online gaming, communicational Internet use, and playing first-person shooters were predictive of externalizing behavior problems (aggression, delinquency).

## Results

### Neurobiological approach to aggression and IAD

*Brain circuitry.* The processing of aggression was summarized by Siever (Siever, 2008) as following: 1) Aggression can be conceptualized as an imbalance of the “top-down” control provided by the prefrontal cortex (e.g. the orbital frontal cortex and anterior cingulate cortex), which is involved in modulating or suppressing aggressive behavior with negative consequences. 2) Aggression can also be explained in terms of excessive “bottom-up” “drives” triggered by the limbic regions such as the amygdale and insula. This concept has been supported by several imaging studies. For example, a number of studies revealed that the prefrontal-limbic circuitry both in structure and function are changed in the specific brain regions in individuals with recurrent aggression or violent behaviors. Volume reduction of gray matter in the prefrontal cortex such as the anterior cingulate and orbito-frontal cortex has been reported in individuals with aggression (Ducharme et al., 2011; Matsuo et al., 2009). A few studies reported that aggression is related with volume reduction of the striatum and amygdale (Ducharme et al., 2011; Schmahl, Vermetten, Elzinga & Douglas Bremner, 2003). Moreover, amygdala and capsule may potentially be indirect neural substrates of aggression, which is evidenced by neurosurgeries. After amygdalotomy, patients showed a reduction in the number of aggressive outbursts (Kim & Lee, 2008; Lee et al., 1998). In another neurosurgical study, combined bilateral anterior capsulotomy and cingulotomy reduced aggressive behavior (Jimenez-Ponce et al., 2011). Other studies also showed that impulsive aggressive individuals showed significant metabolic changes in the orbito-frontal cortex (Rubia et al., 2005; Siever et al., 1999), anterior cingulate cortex (Soloff, Meltzer, Greer, Constantine & Kelly, 2000), insula (Kramer, Riba, Richter & Munte, 2011; Siever et al., 1999) and amygdale (Minzenberg, Fan, New, Tang & Siever, 2007; Passamonti et al., 2012).

Several investigators have tried to clarify specific neural substrates of IAD through structural and functional imaging studies. Among the few studies on gray matter changes in IAD, Zhou et al. (2011) illustrated that individuals with IAD had lower gray matter density in the left anterior cingulate cortex, left posterior cingulate cortex, left insula, and left lingual gyrus. A study by Yuan et al. (2011) showed decreased gray matter volume in the bilateral dorsolateral prefrontal cortex (DLPFC), supplementary motor area, orbito-frontal cortex, cerebellum and left rostral anterior cingulate cortex in the group of subjects with Internet game addiction (Yuan et al., 2011). In addition to gray matter changes, white matter changes have also been observed in IAD. Diffusion tensor image analysis of the Internet game addiction group revealed increased fractional anisotropy (FA) value, indicating greater white matter integrity in the left posterior limb of the internal capsule, and decreased FA value, indicating lesser white matter integrity in the right parahippocampal gyrus (Yuan et al., 2011). Dong et al. (2012) reported that subjects with Internet game addiction showed increased FA in the thalamus and left posterior cingulate cortex compared with healthy controls. Another WM density study presented that subjects with IAD had significantly lower FA values than controls in the orbito-frontal white matter, corpus callosum, cingulum, inferior fronto-occipital fasciculus, and corona radiation, internal and external capsules (Lin et al., 2012).

Several functional magnetic resonance imaging studies are used to survey functional changes in the brain of IAD. Feng et al. (2013) reported that subjects with Internet game addiction showed increased resting cerebral blood flow in the left inferior temporal lobe/fusiform gyrus, left parahippocampal gyrus/amygdala, right medial frontal lobe/anterior cingulate cortex, left insula, right insula, right middle temporal gyrus, right precentral gyrus, left supplementary motor area, left cingulate gyrus, and right inferior parietal lobe. Moreover, decreased cerebral blood flow was also found in the left middle temporal gyrus, left middle occipital gyrus, and right cingulate gyrus. These results showed that IAD could share the core neural substrates of aggression such as the amygdala, insula, and anterior cingulate. Zhou et al. (2011) showed that the right orbitofrontal cortex, right nucleus accumbens, bilateral anterior cingulate and medial frontal cortex, right dorsolateral prefrontal cortex, and right caudate nucleus were activated in the IAD group in contrast to the control group (Ko, Liu et al., 2009). The results by Dong, Huang and Du (2011) reported that IAD was associated with increased activation in the orbitofrontal cortex in gain trials and decreased anterior cingulate activation in loss trials compared with normal controls. They subsequently examined the processing of reward and punishment in IAD and the results showed that individuals with IAD subjectively experienced monetary gain and loss during the performance of a guessing task. Du et al. (2011) showed that the IAD group had increased activation in the right superior parietal lobule, right insular lobe, right precuneus, right cingulate gyrus, and right superior temporal gyrus. The results by Ko et al. (2011) showed that the bilateral dorsolateral prefrontal cortex, precuneus, left parahippocampus, posterior cingulate and right anterior cingulate were activated in response to gaming cues in the Internet game addiction group. When subjects with Internet game addiction saw the pictures related to their addicted game, some brain areas, namely the dorsolateral prefrontal cortex, bilateral temporal cortex, cerebellum, right inferior parietal lobule, right cuneus, right hippocampus, parahippocampal gyrus, and left caudate nucleus showed increased signal activity (Sun et al., 2012).

Through a 18F-fluorodeoxyglucose positron emission tomography study, Park et al. (2010) reported that Internet game over-users had increased glucose metabolism in the right middle orbitofrontal gyrus, left caudate nucleus, and right insula, and decreased metabolism in the bilateral postcentral gyrus, left precentral gyrus, and bilateral occipital regions compared to normal users.

As described above, several structural and functional imaging studies have suggested neural substrates associated with IAD. Interestingly, the key neural substrates of aggression, such as the prefrontal cortex and the limbic system, were included in the brain regions related to IAD. Several studies showed that the neural substrates of IAD could include the key neural substrates of aggression, which explains not only why IAD may frequently accompany aggression, but also why a past diagnosis of aggression could be one of the predictive factors for IAD development (Table 1). Because the two phenomena have a possibility of a common pathological mechanism, they can both be seen as a cause or a result of each other.

### Neuromodulators

*Dopamine and norepinephrine.* The dopaminergic system influences behavioral reinforcement, motivated behavior, and reward processing (Bergh, Eklund, Sodersten & Nordin, 1997; Everitt & Robbins, 2000; Ikemoto & Panksepp, 1999; Kalivas & Volkow, 2005). Addictive disorders commonly display enhanced dopamine levels in the nucleus accumbens and it was enhanced by direct dopamine transmission or by indirect mechanisms that affected dopamine cell firing (Baler & Volkow, 2006). The dopaminergic system also has an important role in the modulation of aggression. In several studies, investigators showed that impulsivity or aggression might be enhanced by dopaminergic hyperfunction (Bergh et al., 1997; Brunner & Hen, 1997; Chotai, Kullgren & Asberg, 1998; Hadfield, 1983; Harrison, Everitt & Robbins, 1997; Miczek, DeBold & van Erp, 1994; Tidey & Miczek, 1996). Furthermore, through pharmacologic challenge studies, manipulation of dopamine levels has been shown to increase or decrease aggression (Brizer, 1988; Chengappa, Ebeling, Kang, Levine & Parepally, 1999; Couppis & Kennedy, 2008; Miczek et al., 1994; Rocca, Marchiaro, Cocuzza & Bogetto, 2002; Schulz, Camlin, Berry & Jesberger, 1999; Schwartzer & Melloni, 2010).

Han et al. (2007) investigated the Taq1A1 allele of the dopamine D2 receptor (DRD2 Taq1A1) and the Val158Met allele in catecholamine-*O*-methyltransferase (COMT) genes in individuals with excessive Internet video game play. Individuals with excessive Internet video game play had an increased prevalence of the DRD2 Taq1A1 and COMT alleles. The study also showed that the DRD2 Taq1A1 allele seemed to be associated with reward dependence in individuals with excessive Internet video game play. A few studies have presented the association between striatal dopamine availability and IAD through measuring the levels of dopamine transporter (DAT) or dopamine D2 receptor. Hou et al. (2012) revealed that the DAT expression level of the striatum was significantly decreased and they suggested IAD is associated with dysfunctions in the dopaminergic brain systems. Kim et al. (2011) showed reduced levels of dopamine D2 receptor availability in the subdivisions of the striatum including the bilateral dorsal caudate and right putamen in individuals with IAD. Although these results could not directly show the dopaminergic hyperfunction of IAD, evidences from these studies suggest that there might be a common mechanism of dopamine system dysfunction in aggression and IAD.

Enhanced noradrenergic receptor sensitivity might be associated with hyper-reactivity to the environment, which could increase the likelihood of aggression (Coccaro et al., 1991). Zhu et al. (2008) observed that electroacupuncture combined with psychologic interference in IAD improved anxiety and the severity of IAD. They also suggested that an improved mechanism is related with a decrease in norepinephrine. Therefore, norepinephrine could be one of the common neuromodulators of IAD and aggression.

*Serotonin*. Biochemical similarities involving serotonin (5-HT) systems have been observed between aggression and IAD. Modulation of the mesolimbic dopamine pathway and serotonergic system dysfunction by the serotonergic system in impulse control disorders reflect inhibitory impairment of the prefrontal cortex (Walsh & Cunningham, 1997). A clinical characteristic of patients with impulsive aggression has been associated with evidence of diminished central serotonergic function in studies of cerebrospinal fluid (CSF) monoamine metabolites. Diminished levels of CSF 5-HIAA are reported in borderline personality disorder patients with violent behavior or impulsive aggression (Brown et al., 1982; Linnoila et al., 1983; Traskman-Bendz, Asberg & Schalling, 1986; Virkkunen, Nuutila, Goodwin & Linnoila, 1987). Pharmacologic challenge studies with serotonergic agonists such as d,l-fenfluramine or ipsapirone have shown a close relationship between aggression and serotonin (Fallon, Keator, Mbogori, Turner & Potkin, 2004; Minzenberg et al., 2006; Siever et al., 1999). Manipulating serotonin via acute tryptophan depletion causes variable effects on brain responses and these studies showed that serotonin had a significant influence on brain regions implicated in aggression (Kramer et al., 2011; Passamonti et al., 2012).

Although few serotonin or serotonin-related gene studies have investigated the question of a possible relationship between serotonin and IAD, Lee et al. (2008) presented that a homozygous short allelic variant of the serotonin transporter gene (SS-5HTTLPR) was more frequent in the excessive Internet use group. Investigators have reported the serotonergic system was important in mediating reward, preference, dependence, and craving of substance addiction (Sari, Johnson & Weedman, 2011; Thompson, Heffner, Strong, Blom & Anthenelli, 2010; Wedekind et al., 2010). More frequent SS-5HTTLPR expressions, which mean increased reuptake of serotonin, are associated with an increase in obsessive–compulsive craving in alcohol dependence (Thompson et al., 2010). Since more frequent expression of the SS-5HTTLPR gene was observed in IAD (Lee et al., 2008), IAD may have a dysfunction of the serotonin system, as observed in previous behavioral addiction studies (Grant, Brewer & Potenza, 2006). Therefore, it is reasonable to assume that IAD is associated with aggression related to serotonin system dysfunction in the aspect of neurobiology.

*Opiates.* Opiates have a close relationship with aggression (Coid, Allolio & Rees, 1983; Goloshchapov, Filipenko, Bondar, Kudryavtseva & Van Ree, 2005; Miller et al., 2004; Symons, Thompson & Rodriguez, 2004), particularly self-directed aggression (Coid et al., 1983; Siever, 2008; Symons et al., 2004). These studies showed a high level of opiate induced self-injurious behavior while opiate antagonists diminished self-injurious acts (Coid et al., 1983; Symons et al., 2004). A study showed that IAD and opiate are related. Bostwick and Bucci (2008) reported a case which showed a successful treatment of an individual with compulsive Internet use with naltrexone, an opioid receptor antagonist. A few researches also suggested naltrexone could play a role in the pharmacotherapy of IAD (Bostwick & Bucci, 2008; Murali & George, 2007).

The opioid system plays a modulatory role on the dopaminergic system in addictive disorders (Ross & Peselow, 2009). Like other addictive disorders, opiates seem to play an important role in IAD. As opiate is one of the most important neuromodulators of aggression along with dopamine or serotonin, IAD with opioid system dysfunction may frequently accompany aggression or violent behaviors.

*Nicotine.* Cholinergic system abnormalities might contribute to limbic hyperactivity and irritability, which can trigger aggression (Siever, 2008). Fallon et al. (2004) reported that hostility traits are related to enhanced susceptibility to nicotine dependence. Low-hostility trait subjects demonstrated no significant changes in brain metabolic response to nicotine administered by patch. High-hostility non-smoker and smoker subjects demonstrated dramatic bilateral metabolic changes to nicotine patch throughout the brain. Cholinergic neurons provide excitatory input to the ventral tegmental area, and sequentially, project from the ventral tegmental area to the nucleus accumbens, resulting in the release of dopamine (Ross & Peselow, 2009). Therefore, nicotinic cholinergic receptors play a role in reinforcing nicotine properties (Heidbreder, 2005). Montag et al. (2012) reported that the nicotinic acetylcholine receptor subunit alpha 4 (CHRNA4) gene could be a candidate gene of Internet addiction. These studies suggest nicotine could be another common neuromodulator of IAD and aggression.

*Other neuromodulators.* In addition to serotonin, dopamine, opiates and nicotine, there are modulators such as glutamate, vasopressin, oxytocin, neurosteroid, sterol and fatty acid that play roles in aggression. However, the association between these neuromodulators and IAD has not yet been investigated. More studies regarding the roles of various neuromodulators are needed.

## Conclusions and Research Directions

IAD is often accompanied with aggression and these two conditions are closely related to each other. Evidences indicate that IAD and aggression have numerous common neural substrates and neuromodulators. Thus, IAD might be a risk factor of aggression and aggression might be a risk factor of IAD (Table 1). Moreover, it is also possible that IAD and aggression have a common neurobiology and impairment in the neural circuitry or in neuromodulator system. These common impairments could lead to the simultaneous development of IAD and aggression. However, no study could definitely describe the neurobiology of IAD, in spite of worldwide attention on IAD. In the DSM-5, IAD is identified as a condition warranting more clinical research and experience before it might be considered for inclusion in the main book as a formal disorder. Many researchers consider pathological Internet use as a form of excessive Internet use, not as behavioral addiction, which deter them from including pathological Internet use in the category of addiction disorders. Therefore, considering the fact that aggression has a relatively definite neurobiology and is a commonly coexisting symptom in various psychiatric disorders, exploring the relationship between IAD and aggression could be one way to establish the identity of IAD. Relatively abundant studies on the relationship between aggression and various psychiatric disorders have been published and contributed to exploration about the neurobiological mechanism of aggression and psychiatric disorders. With more studies on the neurobiology of IAD, the cause of IAD having various psychiatric comorbidities, such as aggressive symptoms, could be elucidated.

The prefrontal and limbic regions and various neuro-modulators were involved in both aggression and IAD. These regions and the neuromodulators were also involved in various psychiatric disorders. Additional evidence-based research is needed to clarify whether IAD can be considered a separate entity in the subset of addiction disorders. IAD is divided into various subtypes (e.g. pornography, game, social networking, blogging, email, Internet shopping, etc.) and have extendability in the future research. Nevertheless, through the exploration of neurobiological similarity between IAD and aggression, we could deal with the aggression of subjects with IAD which drew biopsychosocial attention. Furthermore, we considered the possibility of a separate addiction disorder of IAD and the reason of which aggression could comorbid psychiatric disorder such as addiction disorder.

This article organized the published papers on IAD and showed that aggression was one of the common concomitant symptoms of IAD (Table 1). However, these results do not mean that aggression is due to comorbid IAD. We could suggest that the common coexistence of two phenomena might be caused by biologically shared neural substrates or neuromodulators. More studies regarding the neurobiology of IAD should be conducted.

Internet behaviors of IAD include news searching, web surfing, online gaming, online chatting, adult sex web viewing, online gambling and online studying. Recently, IAD have extended its effect to smart phone addiction. The diversity of the contents of Internet addiction makes research about the relationship and neurobiology between aggression and IAD more difficult but interesting. For example, Internet game addiction does not only encompass Internet addiction but also includes game addiction. In addition, Internet gambling addiction not only includes Internet addiction but also gambling addiction. Therefore, investigators should research the effect of the Internet, game and gambling separately. Indeed, Internet addiction has a complex characteristic.

A number of studies have presented a close relationship between video gaming and aggression, which are summarized through meta-analysis by Anderson et al. (Anderson et al., 2010). Many investigators have shown interest in this relationship between video gaming and aggression and as a result, some researchers have presented that specific gaming characteristics such as competitiveness, difficulty and pace of action could have an influence on aggression related with video game addiction (Weinstein & Lejoyeux, 2010). In the case of Internet game addiction, the difference between Internet game addiction and video game addiction, such as the effect of online, should be further studied. IAD, independently of contents, have been implicated in one area of addictive disorder because human being-continued behaviors such as exploring, communicating and studying could have a strong addictive tendency through engagement with the Internet. It would be interesting and important to investigate how this new psychiatric phenomenon, Internet addiction, affects various psychiatric symptoms including aggression and to reveal the underlying neurobiological mechanism of IAD.

Aside from the huge impact of online activities on people’s lives, IAD should be explored as an important area of behavioral addiction.
